# Method for Accurate Detection of Amino Acids and Mycotoxins in Planetary Atmospheres

**DOI:** 10.3390/life12122122

**Published:** 2022-12-15

**Authors:** Sigrid Madzunkova, Dragan Nikolić

**Affiliations:** 1 La Cañada High School, 4463 Oak Grove Dr, La Cañada Flintridge, CA 91011, USA; 2 California Institute of Technology, Jet Propulsion Laboratory, 4800 Oak Grove Drive, Pasadena, CA 91109, USA

**Keywords:** mass spectrometry, deconvolution, electron impact ionization, collision induced dissociation

## Abstract

We present a systematic analysis of a large number of mass spectra accumulated as the number of ion fragments recorded in unit mass-to-charge detector channels. The method retrieves the abundances of detected species using an efficient deconvolution algorithm, which relies on fragment pattern recognition, mass calibration, and background correction. The abundance analysis identifies target species, amino acids, and mycotoxins through their characteristic fragmentation patterns in the presence of an increasing number of interfering species. The method offered robust and efficient retrieval of abundances of metabolic molecules in complex mixtures obscured by a wide range of toxic compounds.

## 1. Introduction

The in situ exploration of organic environments in the Solar System [1] as signatures of life outside the boundaries of Earth is the most recurring scientific objective in past [2,3,4], recent [5], and future missions [6,7,8]. The search for traces of organic life within our Solar System relies on identifying the building blocks of life, proteins, which are directly linked to the synthesis of amino acids by living organisms [9]. These bio-signatures are not expected to be abundant for most target planets [10,11,12]. Thus, instruments used to investigate them must be sensitive enough to distinguish them unambiguously. In addition, identification through the signature of life amidst organic matter is a difficult task due to sampling site selection [13], biological chirality [14], and molecular diversity [15,16].

Mass spectrometers (MSs) are the instruments of choice for in situ composition analysis of planetary atmospheres and planetary body exospheres [17]. These instruments are classified as ion MSs and neutral MSs, depending on the charge state of the analyzed molecules. Ion MSs rely on studied molecules being electrically charged external to the MS sensor, whereas neutral MS generates ions internally. Both MS types, regardless of ionization methods, differentiate ions by their respective mass-to-charge ratio. Contrarily to the Earth-bound MS, their spaceflight counterparts are much smaller. Scientific payloads comprise less than 10% of the total spacecraft weight. Mass spectrometers are subject to the ever-present need to reduce their sizes by orders of magnitude relative to state-of-the-art laboratory instruments. The scientific requirements often balance this scale-down to retain most analytical capabilities.

This study will not discuss the main differences between MSs in detail, nor will it address the sample collection and preparation methods. Our starting point is the sample as neutral gas, at a given temperature, within an MS. This is a realistic scenario of any current and future investigation where one measures the mass-to-charge ratio signature of an unknown sample and tries to identify or untangle all the species within—all while keeping in mind that in space-exploration scenarios the sample is minute, the time allotted is limited, and the experiment/measurement is performed in preprogrammed sequences. In space-type operations, the data transfers are limited, and MSs generates a large amount of data. Thus, in-situ real-time data processing and data reduction capabilities are very important.

For the ionization method, we assume electron impact ionization and collision dissociation are the most important part of understanding our approach. Namely, each molecule under electron impact ionization will dissociate into a precise pattern (relative abundances between daughter ion fragments) which is a “fingerprint” of a molecule. If one is to match those fingerprints, identification of the parent molecule can be made. Note that there are other ways of identifying unknown species. For example, the high-resolution approach is a very powerful mechanism where ions are determined by their precise mass-to-charge ratio. This method usually focuses on a well-defined mass-to-charge ratio unique only and always to that particular species. Such instruments are widely used, with Orbitrap [18] being one of the most famous. Although the Orbitrap-type instrument is the Holy Grail of laboratory-based mass identification, it is a very complex instrument and challenging to implement for space flight, with LAb-CosmOrbitrap [19] being the latest space instrument prototype in active development. Instrument sensitivity suffers from lower detection efficiency by relying on only one mass channel, where longer scan times needed for the high mass resolution can lead to insufficient data points for fast eluters to define a chromatographic peak [20].

Furthermore, one loses sensitivity by not looking at the full “fingerprint” but at just one specific ion fragment. For example, the amino acid valine has 47 ion fragments that uniquely identify it. Also, the high-resolution modes of operation for any MS are more demanding on measurement time and require higher instrument power consumption. This study is dedicated to the post-measurement analysis of an unknown spectrum through the “fingerprint” detection of postulated species. This method can be used as an analytical tool to decipher complex mixtures detected by other mass spectrometers. The computer programs for analysis [21] were designed for the QITMS instrument developed by JPL, which is simple to build and an easy-to-operate variant of the original Paul Trap MS [22,23], with sensitivity and accuracy comparable to laboratory-based mass spectrometers [24]. These programs were optimized to run on embedded Linux systems and be fast enough to analyze data in real-time [25]. The subset of this program package is relevant for analyzing the International Space Station (ISS) cabin air composition [26], Enceladus cryo-plumes [27], or liquid mixtures of fatty acids and selected amino acids at Ocean Worlds [28]. However, the code scales as the number of species cubed [21]; thus, for large sets of species and a large number of counts (>10^6^), the slowdown is notable. For example, on average, the original random walk code adds one count per species, starting from zero counts, until modeled spectra match the experimental. In this paper, we present a novel approach in which we speed up the process at least eighty times by estimating the initial solution through the inversion of a similarity matrix (Section 2.2). We investigate the sensitivity and accuracy of the method based on various parameters, including the number of detected ion fragments, the uncertainty of fragmentation patterns, and the complexity of the gas mixtures. We also analyze the method’s robustness in the presence of many interfering confounders (molecules of the same mass as the target molecule but with different chemical structures).

This study focuses on training sets containing twenty essential amino acids relevant for in situ detection on extraterrestrial surfaces [29] and four mycotoxins from terrestrial fungi known to colonize spacecraft and ISS [30,31,32,33]. Some fungi may play an important role in long-term spaceflight missions and human space exploration [34] mainly due to the ability of filamentous fungus spores, e.g., Aspergillus niger, to survive prolonged exposures to space radiation [35]. Avoiding biological contamination of other planetary systems is a crucial part of planning for all extraterrestrial missions [36]. Thus, in the search for life-bearing signatures, it is important to account for the presence of mycotoxins as secondary metabolites produced by fungi that colonize spacecraft surfaces.

## 2. Methodology

Governed by the notion that organisms tend to minimize the metabolic cost of protein biosynthesis and, at the same time, maximize the number of amino acid combinations, Krick et al. [9] deduced relative probabilities $P^{\left(c\right)}$ for twenty amino acids listed in Table 1 contained within proteins. These protein-coding amino acids are indexed as target compounds (c) in the first column of Table 1. Their names with unique three-letter codes in parenthesis are given in the second column (name), followed by NIST [37] mass spectrum identification number (NIST EII#) relevant for species fragmentation due to electron impact ionization (EII). We note that our previous experimental MS/MS study [28] used the combination of the EII and the soft chemical ionization in liquid mixtures of alanine, glycine, methionine, phenylalanine, and serine. The fourth column (formula) is a chemical formula followed by metabolic probabilities (*P*^(***c***)^) with which amino acid is likely to be found in living organisms. These probabilities indicate that the least abundant amino acids are tryptophan and cysteine, whereas leucine is the largest. The last column in Table 1 shows a fragmentation similarity, $A^{\left(c\right)}$, of the given amino acid compared to all others. This descriptor takes values from zero (no common fragments exist in all other amino acids) up to 19 (one less than the total number of investigated amino acids). The upper limit, $A^{\left(c\right)}$ = 19, means that the fragmentation of a given amino acid (c) is identical to all others. 

### 2.1. Generation of Mixture Reference Mass Spectrum

**Figure 1:**
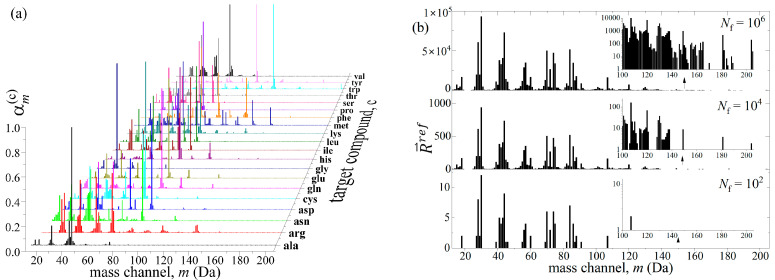
Target compounds listed in Table 1 are characterized by: (**a**) fragmentation probabilities, $\alpha_{m}^{\left(c\right)}$, and their metabolic abundances $P^{\left(c\right)}$ used in the preparation of the reference mass spectrum $\vec{R}{}^{ref}$ shown in (**b**). Insets show increasing complexity of the mass spectra above 100 Da due to the total number of detected ion fragments, $N_{f}$. See text for details.

Each species listed in Table 1 can be uniquely represented as a multi-dimensional vector, $\vec{f}{}^{\left(c\right)}=\underset{m}{\sum }\alpha_{m}^{\left(c\right)}{\vec{e}}_{m}$, where $\alpha_{m}^{\left(c\right)}$ is a measure of likelihood for the compound (c) to contribute an ion fragment of mass m. Eigenvectors ${\vec{e}}_{m}$ are mutually orthogonal (${\vec{e}}_{m}\cdot {\vec{e}}_{m\prime }=0,m\neq m^{\prime })$ and normalized to unity (${\vec{e}}_{m}\cdot {\vec{e}}_{m\prime }=1,m=m\prime$). The fragmentation probabilities, $\pi_{m}^{\left(c\right)}=\alpha_{m}^{\left(c\right)}\cdot \alpha_{m}^{\left(c\right)}$, are derived from the NIST EII database [37] such that the $f^{\left(c\right)}$ is a mass spectrum for the species (c) normalized to unity dot product ($\vec{f}{}^{\left(c\right)}\cdot \vec{f}{}^{\left(c\right)}=\underset{m}{\sum }\pi_{m}^{\left(c\right)}=1$). All unpopulated mass channels m within the given species, are assigned zero fragmentation weight, $\alpha_{m}^{\left(c\right)}=0$. For example, only asparagine contributes the ion fragment m = 24 Da, and none of the amino acids have fragments in the 20 ≤ m ≤ 23 Da mass range. The global mass range is selected to contain all masses between the minimum and the maximum populated mass channel within the given set of compounds, which in this study is from 12 to 206 Da. The illustration of $\vec{f}{}^{\left(c\right)}$ spectra at mass resolution ∆m=1 Da for all twenty amino acids is shown in Figure 1a. These are used in deciding if an ion fragment will be added to a particular mass channel m or not, in an automated procedure using the TrapParticle module from the CITA package [21]. We provide $\vec{f}{}^{\left(c\right)}$ spectra and a prescribed number of fragments $N_{f}$ as input to the TrapParticle module. Here we describe a decision procedure on how to distribute a prescribed number of fragments $N_{f}$ originating from any single species (c) in Table 1 over the global mass range 12 ≤ m ≤ 206 Da.

For each mass channel, *m*, we find fragments that contribute to it, m, we find fragments that contribute to it, $\pi_{m}^{\left(c\right)}$> 0, and we draw a uniform random number, *r*, with values between 0 and 1. If *r*$\leq \pi_{m}^{\left(c\right)}$, we assign one ion fragment from the compound (c) to this mass channel and store it in the “ion cloud” format, which uniquely describes the sampling time, mass (m) and charge (q), position vector, thermal velocity vector, and its compound ancestry (c). We repeat the process until the total number of fragments, $N_{f}$, is distributed over the global mass range, 12 ≤m≤ 206 Da. For a large number of fragments, $N_{f}>10^{6}$, even least-probable ion fragments, for which $\pi_{m}^{\left(c\right)}<10^{-6}$, may appear in the sampled “ion cloud” if the condition $N_{f}\cdot \pi_{m}^{\left(c\right)}>1$ is satisfied. The “ion cloud” is then binned into ∆m=1 Da wide bins concerning their mass-to-charge ratio m/q such that the number of ion fragments, $N_{m}^{\left(c\right)}$, contained in the given mass channel m, constitutes a histogram that we call a single-component reference mass spectrum, $\vec{R}{}_{ref}^{\left(c\right)}$. Thus, a single-component reference mass spectrum, $\vec{R}{}_{ref}^{\left(c\right)}$, can be expressed as, $\vec{R}{}_{ref}^{\left(c\right)}=\cdot \underset{m}{\sum }N_{m}^{\left(c\right)}{\vec{e}}_{m}$, such that it contains the total number of ion fragments, $N_{f}=\cdot \underset{m}{\sum }N_{m}^{\left(c\right)}$, distributed randomly: any subset of ion fragments from the reference mass spectrum, $\vec{R}{}_{ref}^{\left(c\right)}$, will have the same distribution of masses over the global mass range, 12 ≤m≤ 206 Da. Due to its statistical nature, no two “ion clouds” of the same size $N_{f}$ are distributed in the same way within the reference mass spectrum $\vec{R}{}_{ref}^{\left(c\right)}$ since the number of fragments $N_{m}^{\left(c\right)}$ in any of mass channels m has statistical uncertainty $\pm \sqrt{N_{m}^{\left(c\right)}}$. The procedure mentioned above for creating the reference mass spectrum for a single compound (c) can be expanded for arbitrary compound mixtures, $\vec{R}{}^{ref}=\underset{c}{\sum }\eta_{ref}^{\left(c\right)}\vec{R}{}_{ref}^{\left(c\right)}$, where $\underset{c}{\sum }\eta_{ref}^{\left(c\right)}=1$, and such that $\eta_{ref}^{\left(c\right)}$ represents the relative abundance of compound (c) in the reference mixture. One example of such a mixture is given in Table 1, where $\eta_{ref}^{\left(c\right)}=P^{\left(c\right)}$ represents the metabolic cost probability that a given amino acid (c) is present in living organisms [9]. The generation of the mixture reference mass spectrum, $\vec{R}{}^{ref}=\underset{m}{\sum }N_{m}{\vec{e}}_{m}$, for a priori known compound abundances $\eta_{ref}^{\left(c\right)}$ is automated by using the TrapParticle tool from the CITA suite of codes [21]. As previously described, for each mass channel, m, we find all compounds (c) that contribute to this mass channel, $\eta_{ref}^{\left(c\right)}\pi_{m}^{\left(c\right)}$> 0, and we draw a random number r from the uniform distribution, 0 ≤ r ≤ 1. If obtained random value r satisfies the r $\leq \eta_{ref}^{\left(c\right)}\pi_{m}^{\left(c\right)}$ condition, we assign to this mass channel one ion fragment due to the compound (c). This procedure is repeated until the reference mass spectrum $\vec{R}{}^{ref}$ contains the prescribed number ion fragments, $N_{f}=\underset{m}{\sum }N_{m}$. 

Figure 1b illustrates how the complexity of the reference mass spectrum $\vec{R}{}^{ref}$ increases with the total number of ion fragments $N_{f}$, when mixtures are created using the relative metabolic abundances, $\eta_{ref}^{\left(c\right)}=P^{\left(c\right)}$, listed in Table 1. Less probable fragments (for m>100 Da) will be suppressed due to the insufficient count statistics when $N_{f}<10^{3}$. The issue of mass spectrum similarity among different compounds is partly due to the unit mass resolution, ∆m=1 Da, where fine differences in masses of neighboring isobars (fragments with similar masses) will disappear when these mass peaks merge into 1 Da wide mass bins. At this low mass resolution, the dissimilarity between compounds is improved by increased counting statistics, $N_{f}>10^{6}$. In this case, all less-probable fragments start appearing in the reference mass spectrum $\vec{R}{}^{ref}$ and contribute to dissimilarity of compounds, as illustrated in Figure 1b insets. Therefore, throughout this study, we repeat the analysis of a large number of mixtures that form the reference mass spectra, $\vec{R}{}^{ref}=\underset{m}{\sum }N_{m}{\vec{e}}_{m}$, each containing increasing number of ion fragments $N_{f}=10^{2}-10^{6}$, distributed in mass channels m with statistical uncertainty $N_{m}\pm \sqrt{N_{m}}$. Since each ion fragment of mass m present in the “ion cloud” mixture has its own ancestry (c), we a priori know how many of them are due to each compound (c), $N_{m}^{\left(c\right)}$, and thus the total number of ion fragments due to each compound (c), $N^{\left(c\right)}=\underset{m}{\sum }N_{m}^{\left(c\right)}$. Furthermore, we also know the total number of ion fragments, $N_{f}=\underset{c}{\sum }N^{\left(c\right)}$, that is present in the reference mass spectrum $\vec{R}{}^{ref}$, and hence we know abundances $\eta_{ref}^{\left(c\right)}=N^{\left(c\right)}/N_{f}$ of each compound that was used to make the mixture.

### 2.2. Deconvolution of Reference Mass Spectrum

The deconvolution consists of evaluating different trial mixtures of candidate compounds, $\vec{R}=\underset{c}{\sum }\eta^{\left(c\right)}\vec{f}{}^{\left(c\right)}$, by successively changing trial abundances, $\eta^{\left(c\right)}$, until sufficient similarity is achieved between $\vec{R}$ and $\vec{R}{}^{ref}$. The success of retrieval of a priori known abundances $\eta_{ref}^{\left(c\right)}$ is measured by absolute errors, $\varepsilon^{\left(c\right)}=\left|\eta^{\left(c\right)}-\eta_{ref}^{\left(c\right)}\right|$, which depends on the total number of fragments $N_{f}$ contained in $\vec{R}{}^{ref}$. In solving the similarity problem between the two mass spectra, $\vec{R}$ and $\vec{R}{}^{ref}$, we minimize the merit function $\Delta R=min\|\vec{R}-\vec{R}{}^{ref}\|{}_{2}$ with respect to the trial abundance, $\eta^{\left(c\right)}$, by using the iterative constrained least-square random walk method [38]. 

The standard iterative random-walk procedure starts with zeroed initial abundances for each compound, $\eta_{init}^{\left(c\right)}=0$, and successively updates their values $\eta^{\left(c\right)}\to \eta^{\left(c\right)}\pm \delta$ with the fixed step size δ such that the dissimilarity distance ΔR is at the global minimum. Upon convergence, we report final abundances, $\eta_{final}^{\left(c\right)}$, for each compound in the reference mixture and compute the converged retrieval errors, $\varepsilon_{final}^{\left(c\right)}=100\%\left|\frac{\eta_{final}^{\left(c\right)}}{\eta_{ref}^{\left(c\right)}}-1\right|$, as a decreasing function of the total number of fragments, $N_{f}$:

(1) $$\varepsilon_{final}^{\left(c\right)}=\epsilon_{0}^{\left(c\right)}+\epsilon_{1}^{\left(c\right)}/\sqrt{N_{f}}$$

We repeat the random walk retrieval procedure ten times for several fixed numbers of fragments, $10^{2}\leq N_{f}\leq 10^{6}$, and fit the converged retrieval errors, $\varepsilon_{final}^{\left(c\right)}$, according to Equation (1). Fitting parameters, $\epsilon_{0}^{\left(c\right)}$ and $\epsilon_{1}^{\left(c\right)}$, are reported in Table 2 together with their standard uncertainties.

For example, the retrieval error for cystein (cys) from the reference mass spectrum $\vec{R}{}^{ref}$ with $N_{f}=10^{4}$ fragments is computed as follows: $\varepsilon_{final}^{\left(5\right)}=1.36\left(82\right)+\frac{3063\left(237\right)}{100}=32\left(3\right)\%$. The same error is reduced to 4.42(85)%for retrieval of cystein from the spectrum with $N_{f}=10^{6}$ fragments. It is evident from Table 2 that the mass spectrum with the $N_{f}=10^{4}$ fragments can be deconvoluted to better than 16% for most amino acids except for the cystein, and for increased $N_{f}=10^{6}$ number of fragments, the retrieval accuracy is better than 5% for all amino acids studied here.

**Figure 2:**
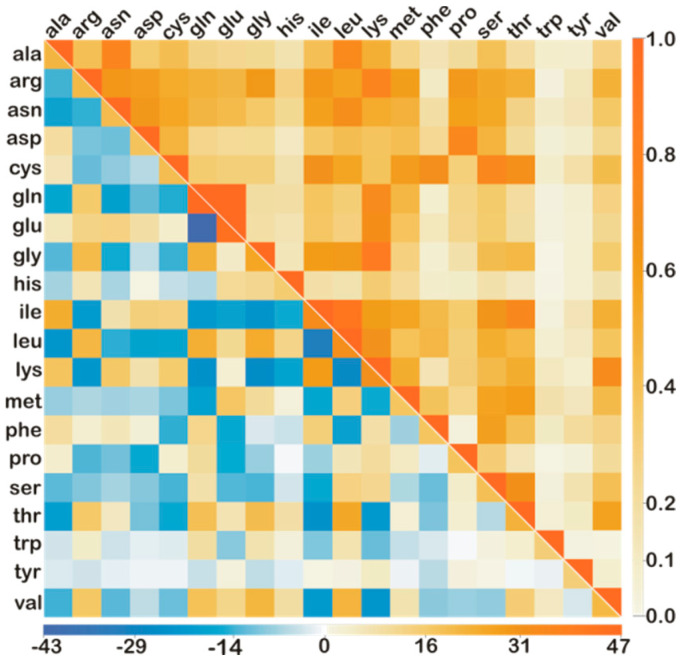
Fragmental similarity matrix [A_c,c’_] (upper triangular part) and its inverse [A_c,c’_]^−1^ (lower triangular part) for the set of twenty amino acids listed in Table 1.

In this study we explored a novel approach to initiate the standard iterative random-walk procedure using an estimate for the initial values of the unknown abundances, such that $\eta_{init}^{\left(c\right)}\neq 0$. For any two compounds, $\left(c\right)$ and $\left(c\prime \right)$, we compute their fragmental similarity value, $A_{c,c\prime }=\vec{f}{}^{\left(c\right)}\cdot \vec{f}{}^{\left(c\prime \right)}=\underset{m}{\sum }\alpha_{m}^{\left(c\right)}\cdot \alpha_{m}^{\left(c\prime \right)}$, and form a symmetric matrix with element values between 0 and 1, as shown in the upper triangular part of Figure 2.

The explicit form for the square of the residual function, $\Delta R^{2}=(\underset{c}{\sum }\eta^{\left(c\right)}\vec{f}{}^{\left(c\right)}-\underset{m}{\sum }N_{m}{\vec{e}}_{m})\cdot (\underset{c\prime }{\sum }\eta^{\left(c\prime \right)}\vec{f}{}^{\left(c\prime \right)}-\underset{m\prime }{\sum }N_{m\prime }{\vec{e}}_{m\prime })$, is minimized with respect to unknown abundances $\eta^{\left(c\right)}$ by finding the first derivatives $\frac{\partial \Delta R^{2}}{\partial \eta^{\left(c\right)}}$ and setting them to zero for every compound (c) in Table 1, which reduces to the following linear system of equations,

$$\sum_{c\prime }\eta_{init}^{\left(c\prime \right)}A_{c,c\prime }=\sum_{m}N_{m}\alpha_{m}^{\left(c\right)},\;\;\;\;\;\;\;\;\;\;\;\;\;\;\;c=1,2,\ldots ,20,$$

or in the matrix form,

(2) $$\left[\begin{array}{ccc} A_{1,1} & \cdots & A_{1,20}\\ \vdots & \ddots & \vdots \\ A_{20,1} & \cdots & A_{20,20} \end{array}\right]\left[\begin{array}{c} \eta_{init}^{\left(1\right)}\\ \vdots \\ \eta_{init}^{\left(20\right)} \end{array}\right]=\left[\begin{array}{c} B_{ref}^{\left(1\right)}\\ \vdots \\ B_{ref}^{\left(20\right)} \end{array}\right]$$

where coefficients, $B_{ref}^{\left(c\right)}=\vec{R}{}^{ref}\cdot \vec{f}{}^{\left(c\right)}=\underset{m}{\sum }N_{m}\alpha_{m}^{\left(c\right)}$, are a priori known for the given mixture of amino acids from Table 1. The largest contributions to the residual function $\Delta R^{2}$ come from fragmentally similar compounds ($0.6\leq A_{c,c\prime }\leq 1$), and to quantify the uniqueness of each mass spectrum shown in Figure 1a, we compute the overlap weights, $A^{\left(c\right)}=\underset{c\prime }{\sum }A_{c,c\prime }$, where $c^{\prime }\neq c$, and list them in the last column of Table 1. Compounds with the smallest $A^{\left(c\right)}$ weights (trp, tir, his, phe, pro) form a core subset for estimating the initial trial abundances, $\eta_{init}^{\left(c\right)}$, to which other compounds can be added as long as Equation (2) remains invertable, as was in this study.

The inverse of the fragmental similarity matrix, ${\left[A_{c,c\prime }\right]}^{-1}$, has element values in the −43 to +47 range, as shown in the lower triangular part of Figure 2. When applied to the column of coefficients, $B_{ref}^{\left(c\right)}$, this inverse matrix yields initial trial abundances $\eta_{init}^{\left(c\right)}$. The $\eta_{init}^{\left(c\right)}$ values are a useful starting point for the multi-dimensional Monte-Carlo random walk simulation algorithm [38,39], which we used here to retrieve the final $\eta_{final}^{\left(c\right)}$ abundances iteratively from reference mass spectra shown in Figure 1b. Namely, by inverting the Equation (2) for the increasing values of the total number of fragments ($N_{f}\leq 10^{6}$), the initial retrieval errors $\varepsilon_{init}^{\left(c\right)}=100\%\left|\frac{\eta_{init}^{\left(c\right)}}{\eta_{ref}^{\left(c\right)}}-1\right|$ remain below 35% for most amino acids listed in Table 1, except for cys (131%), his (74%), met (53%), and trp (185%). Using these initial estimates, $\eta_{init}^{\left(c\right)}$, we proceed with the standard random walk minimization algorithm, which now needs fewer iterations to converge to the same final $\eta_{final}^{\left(c\right)}$ abundances. The overall speedup depends on the iterative step size, δ, and the total number of fragments, $N_{f}$, contained in the reference mass spectrum, $\vec{R}{}^{ref}$. The number of successive updates, $\eta^{\left(c\right)}\to \eta^{\left(c\right)}\pm \delta$, required for the convergence depends on the number of compounds cubed. By using the initial estimates, $\eta_{init}^{\left(c\right)}$, already in the first iteration we accelerate the convergence to within the 35% of the final solution $\eta_{final}^{\left(c\right)}$. Detailed analysis of how the acceleration factor for convergence depends on the number of the compounds used in Equation (2) will be reported elsewhere, and our preliminary findings suggest speedups of at least an order of magnitude for a large number of ion fragments ($N_{f}>10^{6})$. Acceleration of convergence is useful in situations when the chemical composition of atmospheric samples needs to be reported once per second, as is the case onboard the International Space Station, where the QITMS instrument monitors the cabin air composition [25]. 

## 3. Robustness Tests

For extraterrestrial mass spectrometry applications, where the increasing number of interfering species obscures the detection of life-bearing amino acids, the important metric is the robustness of the multi-dimensional Monte-Carlo random walk algorithm [38,39]. Interfering species, hereafter called confounders, are molecules with the same parent mass as the target compounds but may have different fragmentation patterns due to differences in the chemical structure. The simple case of a few target species and a small number of corresponding confounders is given in Table 3. Target compounds (t) are mixed with their respective confounders (c-n) to form the reference mixtures used in deconvolution studies. In a single reference mixture, each confounder enters with the constant unit weight ($\omega_{c-n}$= 1), whereas the corresponding target compound is added according to prescribed weights ($\omega_{t}$= 0, 0.01, 0.02, 0.05, 0.1, 0.2, 0.5, and 1). Every reference mixture is described by its unique ion fragment probability distribution $\vec{R}{}^{mix}$ (mass spectra with a variable number of ion fragments, $N_{f}=$10^3^, 10^4^, 10^5^, or 10^6^)

(3) $$\vec{R}{}^{mix}=N_{f}\cdot (\omega_{t}\vec{f}{}^{\left(t\right)}+\underset{n}{\sum }\omega_{c-n}{\vec{f}{}^{\left(c-n\right)}}^{\left(c-n\right)})/(\omega_{t}+\underset{n}{\sum }\omega_{c-n})$$

which is a weighted sum of individual fragment distributions $\vec{f}{}^{\left(c\right)}$. In this manner, the decision of whether the given mass channel m in the reference mixture mass spectrum $\vec{R}{}^{mix}$ is to be populated by another single ion fragment is governed by a priori known reference probability, $p_{m}^{mix}=(\omega_{t}\alpha_{m}^{\left(t\right)}+\underset{n}{\sum }\omega_{c-n}\alpha_{m}^{\left(c-n\right)})/\left(\omega_{t}+\underset{n}{\sum }\omega_{c-n}\right)$. 

The decision of whether an ion fragment will be added to a particular mass channel m or not is automated using the TrapParticle module from the CITA package [21]. Namely, each accepted ion fragment is stored in the “ion cloud” format, which uniquely describes the sampling time, mass (m) and charge (q), position vector, thermal velocity vector, and its ancestry ((t) for the target or (c-n) for confounder, see Table 3). Due to its statistical nature, no two ion clouds of the same size N_f_ are the same for any prescribed target mixing ratio, $\omega_{t}$. Individual fragment distributions for compounds in Table 3 were generated using canonical SMILES codes as input to the CFM-ID [40] algorithm. 

**Figure 3:**
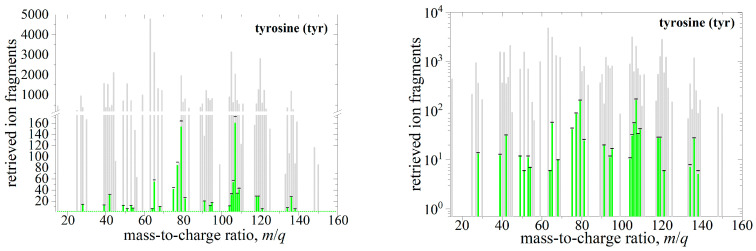
Deconvoluted CID (40 eV) mass spectrum with N_f_ = 51,000 ion fragments due to the mixture of tyrosine and its five confounders shown both in the linear (**left**) and the logarithmic (**right**) scale. The tyrosine reference spectrum is shown in black caps, whereas the retrieved tyrosine spectrum is marked in green bars. The equipartial mixture of interfering confounders and tyrosine in a 1:10 mix ratio is shown in gray bars. See text for details.

CFM-ID predicts Collision Induced Dissociation (CID) fragmentation patterns $\vec{f}{}^{\left(t\right)}$ and $\vec{f}{}^{\left(c-n\right)}$ at 10, 20, and 40 eV relative collision energies, and we use them in Equation (3) to prepare reference mixtures $\vec{R}{}^{mix}$ with equipartial confounders ($\omega_{c-n}$= 1) and variable target weights ($\omega_{t}$= 0, 0.01, 0.02, 0.05, 0.1, 0.2, 0.5, and 1). We then apply a random walk algorithm [38,39] to retrieve the target abundance $\eta^{\left(t\right)}$ and compare them to the reference target abundances contained in $\vec{R}{}^{mix}$. The efficiency of retrieval is illustrated in Figure 3 for the tyrosine (tyr) and its five confounders (c-1, …, c-5). Each confounder is represented by its CID fragmentation pattern $\vec{f}{}^{\left(c-n\right)}$ and mixed equipartially with other confounders resulting in the mass spectrum shown in Figure 3 as grey bars. Consequently, each mass channel belonging to tyrosine is obscured by the contributions of several different confounders. If the reference mixture contained N_f_ = 51,000 ion fragments, then 1023 fragments belonged to tyrosine (1:10 mix ratio to any confounder, i.e., $\omega_{t=tyr}=0.1$). These a priori known reference ion fragments are marked as black caps in Figure 3. The random walk program retrieved 932 tyrosine ion fragments (see green bars in Figure 3) from the reference mass spectrum $\vec{R}{}^{mix}$, which is 8.9% accuracy with 3.3% precision. If the reference mixture contained tyrosine in a 1:100 mix ratio with respect to any confounder ($\omega_{t=tyr}=0.01$), and the mass spectrum contained N_f_ = 501,000 ion fragments (1006 due to tyrosine), the random walk program retrieved 1048 tyrosine fragments—a 4.2% accuracy with 3% precision. 

The retrieval error gets reduced with the improved counting statistics N_f_, which we can illustrate in the example of citrulline and its confounders (third row in Table 3). With a mixture containing N_f_ = 10^6^ ion fragments and 1:1 mixing ratio for citrulline ($\omega_{t=cit}=1$) the random walk retrieved 166,019 out of the initially created 166,490 citrulline fragments, which is 0.28% accuracy with 0.25% precision. 

Similar retrieval accuracies were obtained for other target species found in Table 3. For example, if reference mass spectra contained N_f_ = 51,000 ion fragments and the target mixing ratio was 1:10 to all confounders ($\omega_{t}=0.1$), retrieving accuracies were: 13.2% (pal), 2.9% (arg), 1.1% (lys), 6.1% (orn), 5.8% (gly), and 3.5% (ser). Generating CID fragmentation patterns, $\vec{f}{}^{\left(c-n\right)}$, for increasing number of confounders (n > 10), using CFM-ID [40] algorithm is tedious and requires the knowledge of canonical SMILES codes for each interfering molecule. In that respect, NIST [37] database containing EII (70 eV) fragmentation patterns offers an automated method for compiling a large number of confounders by simply searching the library for the molecules with the same mass as a target compound. 

**Figure 4:**
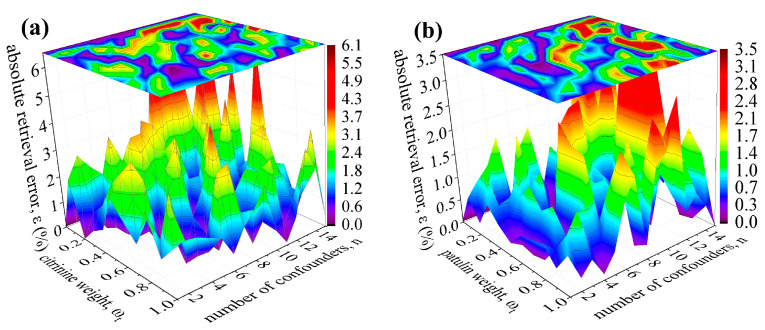

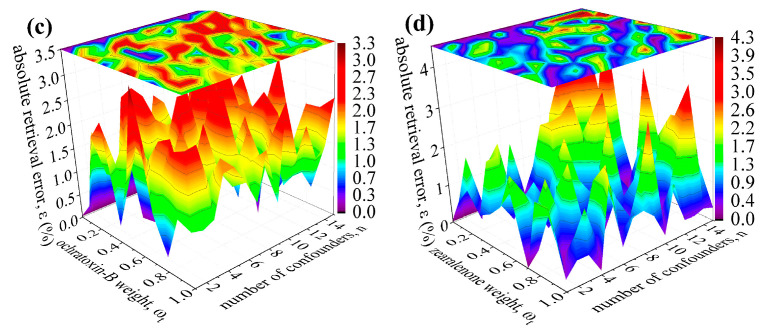
Retrieval accuracy using EII (70 eV) fragmentation patterns to deconvolute mass spectra containing N_f_ = 10,000 ion fragments of mycotoxins and up to 15 confounders. Mycotoxins are: (**a**) citrinin, (**b**) patulin, (**c**) ochratoxin-B, and (**d**) zearalenone. See text for details.

As an example of how well the random walk retrieval algorithm performs against the increasing number of confounders (n > 10), we used EII fragmentation patterns of four mycotoxins: citrinin (NIST#: 241948), patulin (NIST#: 53239), ochratoxin-B (NIST#: 64340), and zearalenone (NIST#: 290624). *Mycotoxins* are fungal secondary metabolites, e.g., fusarium fungi, commonly present as hazardous contaminants in cereal-growing regions [41]. We use them here as representatives of biosignatures recently hypothesized by Limaye and collaborators [42,43] to be dissolved in acidic aerosols that form haze and the cloud layer of Venus. We generated 165 reference mass spectra $\vec{R}{}^{mix}$ for each target mycotoxin as mixtures of up to n = 15 confounders, all present with the unit weights ($\omega_{c-1}=\cdots =\omega_{c-n}$= 1) but with the variable target weights ($\omega_{t}$= 0, 0.1, 0.2, …, 0.9, 1.0). Figure 4 shows that the retrieval method yields no false positives. Namely, when the target mycotoxins are absent from the reference mass spectrum, $\omega_{t}$= 0, the random walk algorithm correctly reports zero abundance, $\eta_{final}^{\left(t\right)}$= 0. As the target mycotoxin reference weight slowly increases towards equipartial mixtures, $\omega_{t}$= 1, retrieval errors remain below 3% only if number of confounders is n ≤ 6, and retrieval errors tend to stay below 6% for n ≤ 15. 

**Figure 5:**
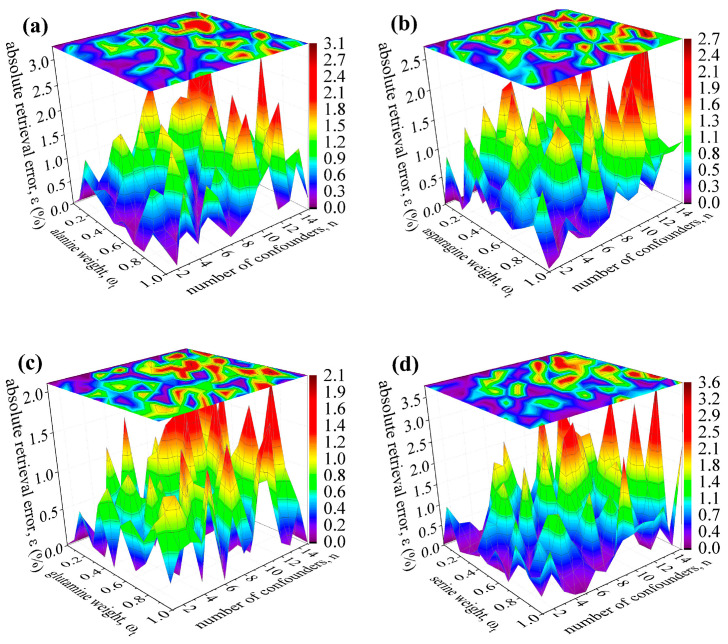
Retrieval accuracy using EII (70 eV) fragmentation patterns to deconvolute mass spectra containing N_f_ = 10,000 ion fragments of amino acids and up to 15 confounders. Amino acids are (**a**) alanine, (**b**) asparagine, (**c**) glutamine, and (**d**) serine. See text for details.

Similar results were obtained for amino acid targets with an increasing number of confounders included in the reference mass spectrum $\vec{R}{}^{mix}$ with N_f_ = 10,000 ion fragments. Figure 5 illustrates that the maximum retrieval errors for alanine, aspargine, glutamine, and serine remain below 3.6%, with the number of confounders n ≤ 15. Further increase in the number of interfering species (n ≤ 24, n ≤ 48, and n ≤ 96) was studied only for the citrulline and the ornithine targets from Table 3 but using their EII (70 eV) fragmentation patterns $\vec{f}{}^{\left(c\right)}$. 

Under these stress conditions, we are using the a priori known fragmentation probabilities $\alpha_{m}^{\left(t\right)}$ for targets when generating the reference mass spectra $\vec{R}{}^{mix}$ with N_f_ = 10^6^ fragments. Targets and confounders were mixed under equipartial conditions ($\omega_{t}=\omega_{c-n}=1$). However, to increase the stress during the retrieval procedure, we enforced an additional 15% random noise on each fragmentation probability $\alpha_{m}^{\left(c\right)}$ belonging only to the confounding species. This modification represents the uncertainty with which potential interfering species are known in advance in extraterrestrial atmospheres. In addition, by randomly perturbing counts in each confounder mass channel m, we introduce the background noise that may be present in the experimental mass spectrum. Results for citrulline show that retrieval error changes from 0.27% for 24 confounders and 0.6% for 48 confounders to 3.8% for 96 confounders. In the case of ornithine, these errors were 0.2%, 5.2%, and 8.9%, respectively. 

**Figure 6:**
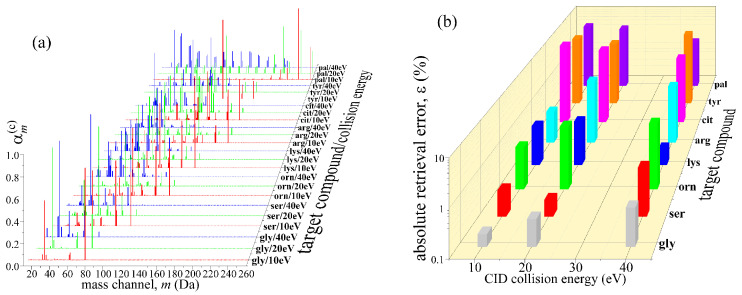
The effect of CID relative collision energies on fragmental distribution and retrieval errors: (**a**) fragmentation probabilities, $\alpha_{m}^{\left(c\right)}$, tend to shift to smaller fragments at 40 eV for most target compounds in Table 3; (**b**) absolute errors remain below 10% when a mass spectrum with 10:1 mixtures contains N_f_ = 51,000 fragment ions.

The fragmentation process of parent molecules differs for different ionization methods. In contrast to the EII, where cross sections for electron ionization at impact energies of 70 eV are standardized, the electrospray ionization (ESI) methods vary. ESI converts solution-phase parent analytes into gas-phase ions, which are then electrostatically extracted into MS at various voltages (10 V, 20 V, and 40 V). Assuming parent gas-phase ions are singly-charged and dependent on 10 eV, 20 eV, or 40 eV kinetic energies, they undergo different fragmentation scenarios in collisions with the solvent vapor and buffer gas (CID), including protonation. Therefore, the distribution of CID fragmentation patterns, as predicted by the CFM-ID [40] tool, changes with the relative collision energies of parent ions and the pressure of buffer gas. The success of the Random Walk retrieval method will always depend on how well fragmentation patterns are known for the given ionization strategy. Most target compounds listed in Table 3 at 10 eV collision energies yield fewer than seven fragments. Still, at 20 eV, additional smaller fragments start to appear, such that at 40 eV number of ion fragments is usually around 30. This behavior is illustrated in Figure 6a, where palmitic acid has fragments over a wide mass range at all three collision energies.

In contrast, ornithine predominantly dissociates in smaller fragments as the collision energy increases. A similar trend is observed for all confounders listed in Table 3, and thus their degree of interference is additionally dependent on the relative collision energy. The absolute error with which Random Walk retrieves the target compound from the 10:1 mixtures of corresponding confounders is shown in Figure 6b. Three largest retrieval errors at collision energy of 10 eV are found for citrulline (9.2%), tyrosine (5.2), and palmitic acid (4.5%). Citrulline also exhibits high retrieval errors at 20 eV (6.9%) and 40 eV (4.3%), followed by tyrosine and palmitic acid (3.9%) at 20 eV, tyrosine (6.9%), and ornithine (3%) at 40 eV. For all other target compounds and collision energies, retrieval errors remain below 3%. Glycine has retrieval errors below 1% mainly because it was obscured by only three confounders and thus was twice as abundant in 10:1 mixtures than other target compounds. The shift of fragment distribution to smaller fragments seen in lysine at 40 eV collision energy improves its retrieval accuracy mostly because its confounders do not follow the similar redistribution, thus making lysine the least similar molecule in the mixture. This is in stark contrast to ornithine and arginine, both of which have retrieval errors between 2.5% and 3.2%, mainly due to a weak propensity for further fragmentation once collision energies exceed 20 eV.

## 4. Summary

We demonstrated applications of a computational method to retrieve relative abundances of amino acids and mycotoxins from complex mixtures containing a large number of interfering species. Obtained results are encouraging and show that life-bearing target species and species that are the product of the metabolism of microbial organisms can be detected with accuracies better than 10% for the sufficient counting statistics and sensitivity readily achievable with modern mass spectrometers. A novel contribution to this study is the method to speed up the convergence times computationally. Speedup comes from the inversion of the fragmental similarity matrix, which provides an optimal starting point for the standard random walk procedure used in previous studies. Future studies will focus on expanding the number of species to include fatty acids and products of microbial metabolism.

## Figures and Tables

**Table 1:** Target compounds (amino acids) used in this study.

(c)	Name	NIST EII #	Formula	$P^{\left(c\right)}$	$A^{\left(c\right)}$
1	alanine (ala)	228084	C_3_H_7_NO_2_	0.076	2.89
2	arginine (arg)	154822	C_6_H_14_N_4_O_2_	0.058	4.90
3	asparagine (asn)	229288	C_4_H_8_N_2_O_3_	0.052	4.52
4	aspartic acid (asp)	230141	C_4_H_7_NO_4_	0.057	3.06
5	cysteine (cys)	228086	C_3_H_7_NO_2_S	0.010	4.42
6	glutamine (gln)	228123	C_5_H_10_N_2_O_3_	0.055	3.31
7	glutamic acid (glu)	228122	C_5_H_9_NO_4_	0.063	3.08
8	glycine (gly)	229287	C_2_H_5_NO_2_	0.076	3.09
9	histidine (his)	228152	C_6_H_9_N_3_O_2_	0.018	0.96
10	isoleucine (ile)	228158	C_6_H_13_NO_2_	0.066	5.18
11	leucine (leu)	228159	C_6_H_13_NO_2_	0.067	4.78
12	lysine (lys)	26152	C_6_H_14_N_2_O_2_	0.040	5.36
13	methionine (met)	191948	C_5_H_11_NO_2_S	0.023	3.66
14	phenylalanine (phe)	107173	C_9_H_11_NO_2_	0.044	2.09
15	proline (pro)	228120	C_5_H_9_NO_2_	0.066	2.43
16	serine (ser)	228085	C_3_H_7_NO_3_	0.065	4.29
17	threonine (thr)	26149	C_4_H_9_NO_3_	0.058	3.64
18	tryptophan (trp)	127959	C_11_H_12_N_2_O_2_	0.007	0.32
19	tyrosine (tyr)	228180	C_9_H_11_NO_3_	0.030	0.54
20	valine (val)	26146	C_5_H_11_NO_2_	0.069	2.90

**Table 2:** Values of fitting parameters for the retrieval errors, see Equation (1), of amino acid abundances listed in Table 1. Standard deviations are enclosed in brackets.

(c)		$\epsilon_{0}^{\left(c\right)}$	$\epsilon_{1}^{\left(c\right)}$	(c)		$\epsilon_{0}^{\left(c\right)}$	$\epsilon_{1}^{\left(c\right)}$	(c)		$\epsilon_{0}^{\left(c\right)}$	$\epsilon_{1}^{\left(c\right)}$	(c)		$\epsilon_{0}^{\left(c\right)}$	$\epsilon_{1}^{\left(c\right)}$
1	ala	0.12(15)	661(62)	6	gln	3.8(7)	878(131)	11	leu	1.83(84)	931(175)	16	ser	0.15(14)	655(51)
2	arg	0.1(3)	1321(132)	7	glu	2.1(3)	798(77)	12	lys	2.6(9)	1302(265)	17	thr	0.2(3)	905(91)
3	asn	0.44(19)	1077(83)	8	gly	0.57(17)	596(46)	13	met	0.22(19)	622(77)	18	trp	0.10(8)	275(28)
4	asp	0.28(15)	497(54)	9	his	0.26(18)	686(54)	14	phe	0.12(7)	514(44)	19	tyr	0.10(7)	249(20)
5	cys	1.36(82)	3063(237)	10	ile	1.88(86)	1286(188)	15	pro	0.15(11)	519(63)	20	val	0.52(35)	628(85)

**Table 3:** List of target compounds and their confounders used in robustness studies.

(t)	Target Name	Target Formula	Confounders (Canonical SMILES)					
			c-1	c-2	c-3	c-4	c-5	c-6
(pal)	palmitic acid	C_16_H_32_O_2_	CCCCC(CC)C(=O)C(O)C(CC)CCCC	CCCCCCCCCCCCC(C)(O)C(C)=O	CCCCCCCCCCC(C)(C(=O)O)C(C)C	CCCC(C)CC(C)CC(CC)(CCC)C(=O)O	CCCCCCCC(CC)(CCCC)C(=O)O	CCCCCCCCCC(C)CC(C)CC(=O)O
(tyr)	tyrosine	C_9_H_11_NO_3_	CC(=O)c1c(C)[nH]c(C(=O)O)c1C	Cc1cc(C)n(C)c(=O)c1C(=O)O	Nc1cc(CCC(=O)O)ccc1O	NCCOc1ccc(C(=O)O)cc1	COc1ccc(NCC(=O)O)cc1	--
(cit)	citrulline	C_6_H_13_N_3_O_3_	[H][C@](O)(CCCNC(=N)N)C(=O)O	[H][C@]1(O)CN(C(=N)N)C[C@@]([H])(O)C1O	COCC(N)C(=O)NCC(N)=O	COC(CN)CC(=O)NC(N)=O	C(CNC(=O)CNC(=O)N)OC	--
(arg)	arginine	C_6_H_14_N_4_O_2_	NC(=O)NCCCCNC(N)=O	NC(=O)CC(N)C(N)CC(N)=O	CC(NCC(N)C(N)=O)C(N)=O	NC(=O)NCCCC(N)C(N)=O	N=C(N)NCCC(O)C(N)C=O	--
(lys)	lysine	C_6_H_14_N_2_O_2_	CN(C)CCCNC(=O)O	COCCNC(=O)[C@@H](C)N	CN(CC(C)(C)O)C(N)=O	CCC[C@H](N)C(O)C(N)=O	--	--
(orn)	ornithine	C_5_H_12_N_2_O_2_	CN(C)CCNC(=O)O	[H][C@](C)(CN)NC(=O)OC	CN(C)C(=O)[C@@H](N)CO	N[C@@H]1COCOC[C@@H]1N	COC[C@@H](C)NC(N)=O	--
(gly)	glycine	C_2_H_5_NO_2_	NC(=O)CO	COC(N)=O	CNC(=O)O	--	--	--
(ser)	serine	C_3_H_7_NO_3_	n1ccccc1C=C	c1(cccnc1)C=C	c1(ccncc1)C=C	C1=CC=C(C=C1)C=N	N#Cc1nccnc1	C(C#N)C(C#N)C#N

## Data Availability

No new data were created or analyzed in this study. Data sharing is not applicable to this article.
